# 710. Burden of Clostridioides difficile Infection (CDI) in Flint, Michigan (MI): Association of Race and Gender with Mortality

**DOI:** 10.1093/ofid/ofad500.772

**Published:** 2023-11-27

**Authors:** Mariam Younas, Hina Amin, Elio Ragheb, Abdullahi Mahgoub, Bibek Karki, Manoj Upadhyay, Mohammad Bakeer, Anthony Armor, Ann Newell, Carlos F Ríos-Bedoya

**Affiliations:** Hurley Medical Center/Michigan State University College of Human Medicine, Flint, Michigan; Hurley Medical Center, Flint, Michigan; Hurley Medical Center, Flint, Michigan; MSU/ Hurley Medical Center, Flint, Michigan; Hurley Medical Center/MSU, Flint, Michigan; Hurley Medical Center, Flint, Michigan; Hurley Medical Center, Flint, Michigan; Hurley Medical Center, Flint, Michigan; Hurley Medical Center, Flint, Michigan; Hurley Medical Center, Flint, Michigan

## Abstract

**Background:**

Flint, MI faces an increased vulnerability to health disparities as compared to other communities in MI. Flint & Genesee County’s most critical health needs is informed upon in-part by data provided by the three community hospitals, serving Genesee County, MI, and its urban core, the City of Flint. This cross-sectional study aims to describe our experience with CDI in Flint, MI; and determine 30-day mortality among CDI cases based on gender and race.

**Methods:**

Hurley is a 443-bed premier public teaching hospital (largest in the region). Using National Healthcare Safety Network (NHSN) surveillance Laboratory Identification (Lab ID) events, 926 CDI cases, ≥ 18 years of age were identified among patients hospitalized at Hurley Medical Center from 01/2015 to 07/2022. A positive stool C. difficile test was regarded as a “CDI case” for purpose of this study. NHSN surveillance definitions were used to describe the incident, recurrent cases and determine the epidemiologic categories.

**Results:**

During the 7.6-year study period, 926 cases were identified. 46.7% of the cases were in men, with a 30-day mortality of 10.4% vs 7.3% in women (p-value 0.10) (Table 1). African American (AA) comprised 44.4% of the cases with 30-day mortality of 9.1% vs 8.1% in whites (p-value 0.78) (Table 2). There were 64 recurrent CDI cases with 30-day mortality of 6.3% as compared to 8.9% in incident cases (p-value 0.65). Mean age at specimen collection was 59.9 years (standard deviation (SD) 17.5) with 43% of the cases falling in the age group ≥ 65 years (Table 3). Hospital-onset (HO) and community-onset healthcare facility associated (CO-HCFA) CDI accounted for 42.8% (396/926) and 14.9% (138/926) of the cases respectively. 60.5% (237/392) of the CO CDI were associated with antibiotic use with in the past 90 days as compared to 95.9% (380/396) of HO and 94.9% (131/138) of CO-HCFA CDI (p-value < 0.001).

**Table 1:**
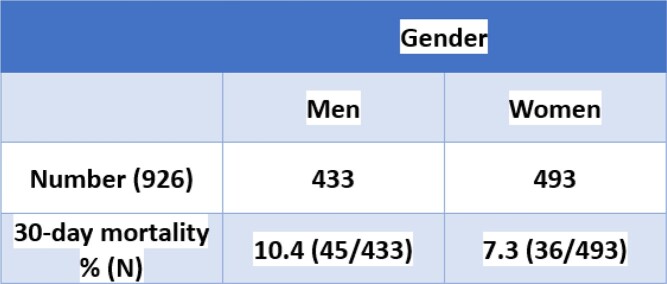
CDI cases based on gender

**Table 2:**
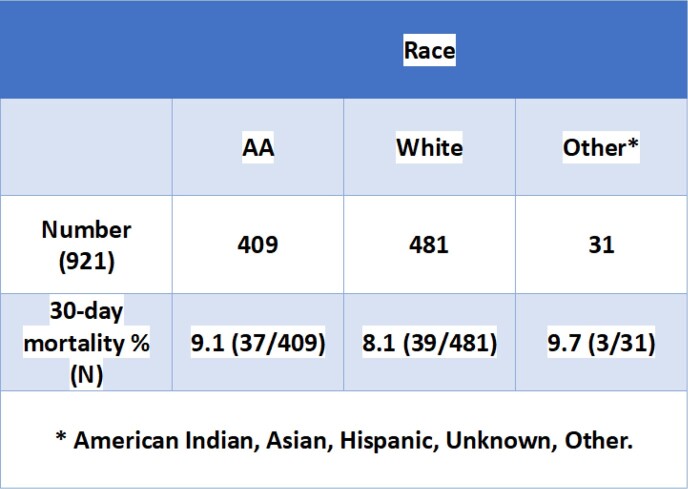
CDI cases based on race

**Table 3:**
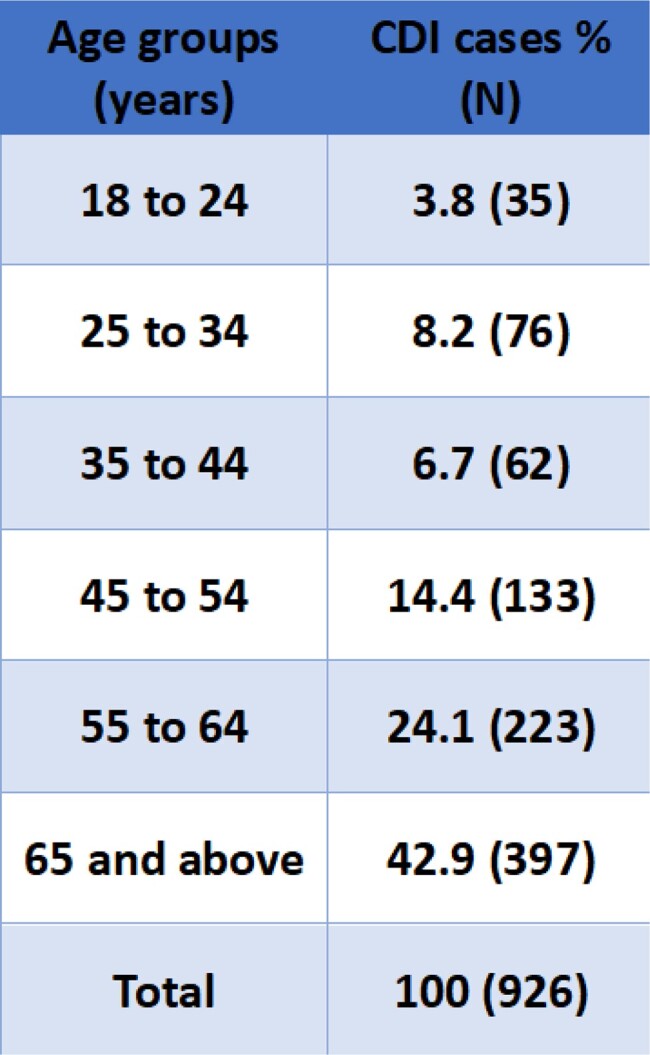
CDI cases based on age groups

**Conclusion:**

CDI caried a significant mortality in this high-risk cohort. Mortality was slightly higher in men and AA; though it was not statistically significant. Antibiotic use was the strongest predictor of CDI acquisition (p-value < 0.001) and about half the CDI cases were CO.

Antimicrobial stewardship initiatives targeting unnecessary and inappropriate antimicrobial use in the community are as imperative as in the hospital setting.

**Disclosures:**

**All Authors**: No reported disclosures

